# The Applicability of Shear Wave Elastography to Assess Myotendinous Stiffness of Lower Limbs during an Incremental Isometric Strength Test

**DOI:** 10.3390/s22208033

**Published:** 2022-10-21

**Authors:** Alfredo Bravo-Sánchez, Pablo Abián, Giacomo Lucenteforte, Fernando Jiménez, Javier Abián-Vicén

**Affiliations:** 1 Performance and Sport Rehabilitation Laboratory, Faculty of Sport Sciences, University of Castilla-La Mancha, 45071 Toledo, Spain; 2 Faculty of Health Sciences, Universidad Francisco de Vitoria, 28223 Pozuelo de Alarcón, Spain; 3 Faculty of Humanities and Social Sciences, Comillas Pontifical University, 28049 Madrid, Spain; 4 Education and Research Department, Isokinetic Medical Group, FIFA Medical Centre of Excellence, 40132 Bologna, Italy

**Keywords:** muscle, tendon, mechanical properties, ultrasound

## Abstract

The aim of the study was to describe the applicability of shear wave elastography to assess muscular and tendinous stiffness of the lower limbs during an incremental isometric strength test and to differentiate the stiffness evolution between superficial and deep muscle regions. Dominant rectus femoris and patellar tendons of 30 physically active people (28.3 ± 9.2 years, 173.2 ± 7.7 cm, 76.2 ± 12.6 kg) were measured in different isometric strength conditions (relaxed muscle, and at 10%, 20%, 30%, 40%, 50% and 60% of maximal voluntary contraction (MVC)). The percentage of success was >85% at all muscle contraction intensities for rectus femoris muscles but only in a relaxed condition for patellar tendons. Rectus femoris stiffness significantly increased compared to the relaxed condition from 30% to 60% MVC (*p* ≤ 0.011) in superficial muscle regions, and from 10% to 60% MVC (*p* ≤ 0.002) in deep muscle regions. Deep muscle regions showed higher stiffness values than superficial muscle regions at 30% MVC (51.46 ± 38.17 vs. 31.83 ± 17.05 kPa; *p* = 0.019), 40% MVC (75.21 ± 42.27 vs. 51.25 ± 28.90 kPa; *p* = 0.018), 50% MCV (85.34 ± 45.05 vs. 61.16 ± 37.03 kPa; *p* = 0.034) and 60% MVC (109.29 ± 40.04 vs. 76.67 ± 36.07 kPa; *p* = 0.002). Rectus femoris stiffness increased during the incremental isometric contraction test, and inter-region differences were found at 30% MVC.

## 1. Introduction

Stiffness is defined as the resistance offered by muscle tissue to deformation against an external force that tries to modify its initial shape [1]. The importance of myotendinous stiffness lies in its influence on the joint stability [2,3] and its relationship to explosive force production [4] and to stretching–shortening cycle activity performance [5]. Furthermore, the assessment of small changes in stiffness can be a method of early identification of the development of muscle fatigue, muscle damage or muscle injury [6,7], adaptations to sport practice [8,9] and even age-related changes in myotendinous properties [10]. The relationship between myotendinous stiffness and sport performance and injury prevention has provoked an increase in the number of studies that aim to describe the stiffness in different conditions [7,11].

The study of the mechanical properties in dynamic exercise started with the use of conventional ultrasound during an isometric contraction, in which the myotendinous stiffness resulted in the combination of structural changes and force production [12,13,14]. This measurement requires great expertise and could sometimes be difficult due to the loss of the reference point of the tissue and the anatomical heterogeneity between participants [14]. Elastography is a new measurement technique commonly used to locate tissues that present an abnormality or disease [15], although it has also been used to describe return-to-play adaptations [16], to describe the tissue mechanical properties in athletes [6,10,13] and to analyze the effect of aging on the stiffness of different muscles [17,18,19], showing good reliability for the assessment of superficial muscles [20]. Using elastography, depending on the mechanical stimulus used to evaluate tissue deformation, we can differentiate between strain elastography and shear wave elastography. Shear wave elastography has been more widely used in research than strain elastography, due to its lower operator dependence and better reproducibility [19,21].

The majority of studies that used shear wave elastography to analyze the mechanical properties of muscles and tendons applied this technique in a relaxed condition, without muscle contraction [6,21], and the investigations that reported dynamic evaluation of the stiffness poorly defined the muscle contraction intensities or the range of muscle contraction intensities was low [22,23]. Soldos et al. [24] described the Young’s modulus of vastus lateralis during a maximal isometric contraction and found greater values in athletes than the control group, although the sample size was small. Commonly, during the shear wave evaluation, a circular region of interest (ROI) is placed inside the muscle belly, which gives an average value of the muscle stiffness but does not allow for differentiation between muscle regions [21], which could give a better explanation of the relationship between myotendinous stiffness values and the behavior of the tissues during muscle contraction. In this context, Koo et al. [25] provided preliminary data of muscle stiffness differentiating between superficial and deep muscle regions during passive stretching, so it is necessary to understand what occurs during muscle contraction. Perhaps the main problem during dynamic studies would be the saturation of the elastogram, described as the signal loss and collapse of the ultrasound analysis due to the stiff condition of the evaluated tissue [26]. The saturation of the elastogram occurs when the elasticity of the evaluated tissue exceeds the upper limit of the elasticity that the equipment can measure [26]. Therefore, it is necessary to describe the range of muscle contraction intensities where shear wave elastography is applicable.

Nowadays, the elastography exams of muscles and tendons seem to be limited to basal conditions; therefore, the main objective of our research was to analyze the applicability of shear wave elastography to assess the myotendinous stiffness of the lower limbs during an incremental isometric strength test. As a secondary objective, we aimed to describe the difference in stiffness evolution between superficial and deep muscle regions during the incremental isometric test.

## 2. Materials and Methods

Thirty physically active males were recruited for the study (28.3 ± 9.2 years, 173.2 ± 7.7 cm, 76.2 ± 12.6 kg, 18.2 ± 4.6% fat). Before the measurements, all subjects signed an informed consent form. The Ethics Committee of Clinical Research at the Toledo Hospital complex approved the study. Participants with an injury or any pain that would prevent them from completing their usual physical activity were excluded from the sample. All data were recorded on the dominant lower limb, which was indicated by the participants before data collection, via a questionnaire, as the self-reported preferred lower limb for kicking [27].

### 2.1. Design and Procedure

A descriptive study was carried out to determine the applicability of shear wave elastography during a dynamic exam. Each participant undertook the testing procedure, consisting of seven stiffness measurements, on the same day, with different intensities of muscle contractions. All the participants were evaluated on their rectus femoris and patellar tendon of the dominant lower limb, and the shear wave evaluator was blinded to the muscle contraction intensity. To evaluate the intensity range of muscle contraction, a maximal strength test was carried out before elastography assessment. Participants completed a day in the laboratory during which they familiarized themselves with the measurement protocols three days before the data collection process.

The measurement points and the position of the patients during the exams were standardized. Ultrasound exams were performed at 50% of the distance from the upper pole of the patella to the anterior superior iliac spine for the rectus femoris and 1 cm cranial to tibialis tuberosity for the patellar tendon. Participants were sitting with 110° of hip flexion and 20° of knee flexion during all the exams. Each subject completed a familiarization process with the experimental procedures the week before the data collection.

### 2.2. Isometric Muscle Contraction Work

After a 5 min warm-up at submaximal intensity of knee flexion–extension movement, participants performed a maximal voluntary contraction force (MVC) test. MVC was assessed with an isokinetic dynamometer (Biodex System 3; Biodex Medical Systems, Inc., Shirley, NY, USA). The test was carried out after completion of 2 repetitions of isometric muscle contractions (knee extension) of a 5 s duration with a 60 s recovery period between repetitions. The dynamometer lever arm was attached 1–2 cm cranially to the lateral malleolus with a Velcro strap. The lateral femoral condyle and the dynamometer axis of rotation were aligned, and participants were secured firmly to the dynamometer seat with Velcro straps across the chest and hips and were instructed to grip the seat to stabilize the pelvis during the muscle contractions. The absolute MVC peak torque was determined as the peak force reached during maximal efforts and was defined as the reference value with which to calculate the intensities of submaximal contractions [6]. To measure the stiffness of the rectus femoris and patellar tendon in dynamic conditions, participants carried out submaximal muscle isometric contractions of a 5 s duration during a knee isometric extension assessment. The range of muscle contraction intensity varied from 10 to 60% MVC, divided into incremental steps of 10% MVC (10% MVC, 20% MVC, 30% MVC, 40% MVC, 50% MVC and 60% MVC). Feedback of muscle intensity contraction was shown to the participants during the test, and they were required to keep the effort line at the intensity mark showed on the device screen. All the participants were randomly tested twice at each intensity of muscle contraction. Between each muscle contraction, a 60 s rest period was applied, and between sets of contractions (from 10 to 60% MVC in a random order), there was a 180 s rest period.

### 2.3. Shear Wave Elastography Assessment

**Figure 1:**
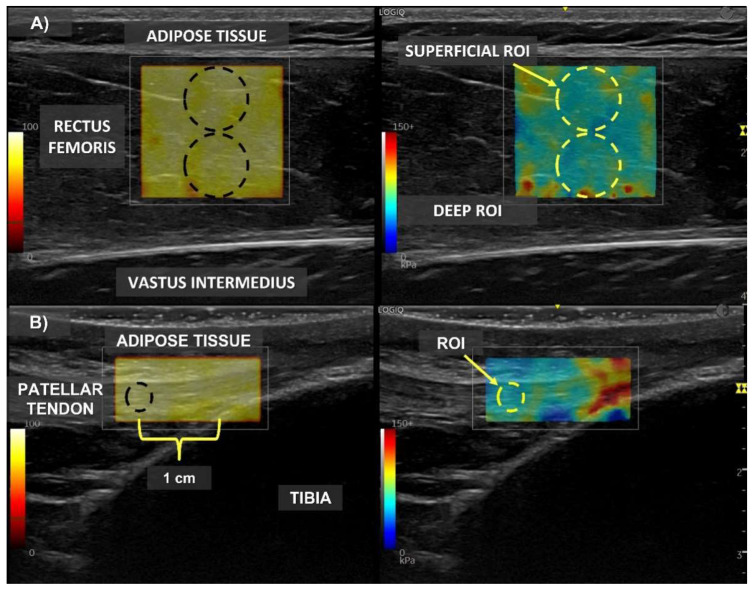
Example of elastography measurement. (**A**) Shear wave elastography measurement of rectus femoris; (**B**) shear wave elastography measurement of patellar tendon; E = shear modulus; ROI = region of interest.

The shear wave elastography exam was performed with a Logiq^®^ P8 ultrasound (GE Healthcare, Milwaukee, WI, USA) with a 2–11 MHz multifrequency linear probe L3-12-D (GE Healthcare system, Milwaukee, WI, USA). The ultrasound probe was aligned with the rectus femoris fascicles or with the longitudinal axis of the patellar tendon, and it was placed with very light pressure on top of the gel. The B-mode image and elastogram color map were displayed side-by-side on the screen during elastography measurements. To avoid anisotropy of the under-probe tissue, (a) the examination probe was held perpendicular to the tissue and (b) the B-mode image showed continuous striations (muscle fascicle) extending from superficial to deep aponeurosis [28]. Four permanent waterproof skin landmarks were drawn with a marker under 2D-mode monitoring during the pre-session to keep the same probe location during all the measurements [7]. To assess the stiffness of the rectus femoris and to differentiate between the superficial and deep muscle regions, two circular ROIs were set inside the muscle belly with the mid-distance of muscle thickness in diameter and without connective tissue inside them (Figure 1). For the patellar tendon assessment, a single circular ROI was placed inside the tendon structure with tendon thickness size in diameter (Figure 1). The stiffness was evaluated after 5 s acquisition exams for all the muscle contraction intensities described above, trying to keep the real-time color map as homogeneous as possible. In addition, before the warm-up, the participants were evaluated in relaxed conditions (without muscle contraction), and this measurement was employed as a basal reference. All the elastography exams were performed by the same expert, who had more than 20 years’ experience in sports medicine and ultrasound application (F.J.).

### 2.4. Statistical Analysis

The statistical analysis was performed with IBM SPSS Statistics 26.0 (SPSS, Chicago, Illinois). All data were expressed as mean ± standard deviation (SD). The data were tested for normality with a Shapiro–Wilk test, and since the assumption of normality (for all variables *p* > 0.05) was verified, the significance of differences between muscle region (superficial or deep region) and strength condition (from relaxed muscle to 60% MVC) were calculated with two-way ANOVA. The success of the shear wave application during the evaluation of rectus femoris and patellar tendon stiffness was defined as the evaluation images in which no saturation of elastogram was found. The percentage of success of shear wave application in rectus femoris and patellar tendon analysis was calculated as (1):

Success percentage (%) = Number of successful measurements/Number of participants × 100(1)

The number of successful measurements was described as the number of exams in which the elastogram was homogeneous and there was no saturation inside of the ROI. The level of significance was *p* < 0.05.

## 3. Results

Table 1 shows the results of the elastography evaluation of the patellar tendon and rectus femoris and the percentage of success of the measurement in relation to the intensity of muscle contraction. A success rate > 85% was achieved in all the conditions studied for the rectus femoris. The stiffness of the patellar tendon was successfully described for all participants in the basal condition (82.37 ± 27.97 kPa), and 70% success was achieved at 10% MVC (186.24 ± 36.87 kPa). For 20% MVC, the success percentage was 40%, for 30% MVC and 40% MVC the success percentage was 30%, and for 50% MVC the success percentage was 10%. The success rate was 0% to 60% MVC.

**Figure 2:**
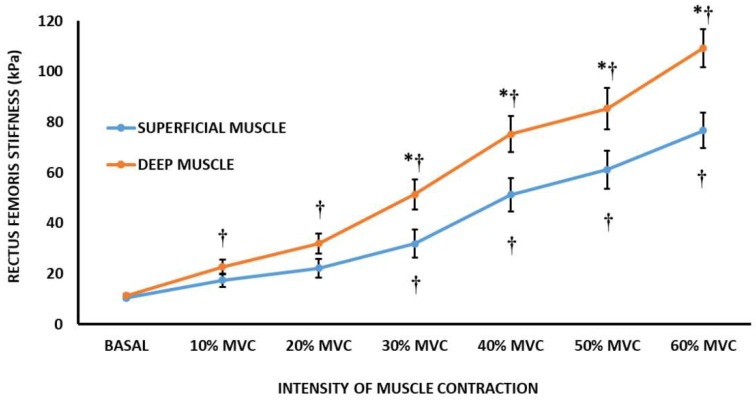
Rectus femoris stiffness during an isometric strength test at different levels of muscle contraction (mean ± SD). Basal = basal condition; MVC = Maximal voluntary contraction force; 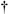 Differences to basal condition. ^﹡^ Differences between superficial muscle region and deep muscle region; *p* < 0.05.

Figure 2 shows the evolution of the rectus femoris stiffness from the basal condition to 60% MVC. The stiffness of rectus femoris was significantly higher compared to the basal condition from 30% MVC to 60% MVC (*p* = 0.011) in the superficial muscle region. In the deep muscle region of the rectus femoris, the significant increase in stiffness compared to the basal condition occurred at a lower level of muscle contraction intensity, 10% MVC, and remained until the end of the incremental test (*p* = 0.002). In addition, in the muscle region comparison, deep muscle regions showed higher stiffness values at 30% MVC (51.46 ± 38.17 vs. 31.83 ± 17.05 kPa; *p* = 0.019), 40% MVC (75.21 ± 42.27 vs. 51.25 ± 28.90 kPa; *p* = 0.018), 50% MCV (85.34 ± 45.05 vs. 61.16 ± 37.03 kPa; *p* = 0.034) and 60% MVC (109.29 ± 40.04 vs. 76.67 ± 36.07 kPa; *p* = 0.002) than superficial muscle regions.

## 4. Discussion

The main objective of our study was to evaluate the applicability of shear wave elastography for measuring myotendinous stiffness in dynamic conditions. In addition, we described the evolution of the rectus femoris during an incremental isometric strength test differentiating between superficial muscle tissue and deep muscle tissue. Although the percentage of success was 100% for the elastography assessment in the basal condition, elastography was not able to assess the stiffness of the patellar tendon from 10% MVC with a success rate lower than 50% for higher contraction intensities. Therefore, when the tissue stiffness exceeded 200 kPa, it seemed to cause saturation of the elastogram, which led to the invalidation of the measurement technique. On the other hand, the stiffness of the rectus femoris muscle increased significantly compared to the basal condition from a contraction intensity of 10% MVC to 60% MVC, and in particular, the stiffness of the deep region of the rectus femoris increased more than that of the superficial region from 30% to 60% MVC. This difference related to the depth of the ROI position during the measurement should be taken into account by investigators in future research.

Elastogram saturation [26] is one of the main problems occurring in the use of elastography for evaluating the mechanical properties of tissues, even in basal conditions without muscle contraction. The elastogram saturation implies that the probe is not capable of interpreting the waves of ultrasound-generated noncolored spaces within the elastogram from which data cannot be obtained [29]. When saturated areas are large, as occurred in patellar tendons in our study, it is difficult to be objective during measurement with elastography because they should not be included in the ROI. In addition, these noncolored areas reduce the reproducibility of the elastography technique. Our results show that for the dynamic evaluation of patellar tendon stiffness, it would be necessary to improve the capability of ultrasound probes, taking into account that we only achieved 40% success during the measurement at 20% MVC and a lower percentage of success when the intensity of the contraction was greater. This saturation problem has already been reported by Bravo-Sanchez, Abian, Sanchez-Infante, Esteban-Gacia, Jimenez and Abian-Vicen [21], who found some difficulties in the assessment of the vastus lateralis in basal conditions when the superficial connective tissue had a big thickness, reaffirming the need to improve the technology in this aspect.

This investigation is one of the first studies to apply shear wave elastography during a dynamic assessment of rectus femoris stiffness and according to previous studies, where rectus femoris stiffness was the result of combining tissue deformation measured with ultrasound in B mode and the muscle force produced during an isometric contraction [30]; the stiffness of the rectus femoris measured with elastography increased significantly with the intensity of the contraction. The increase in the muscle stiffness that we observed in our study was ~7 times greater compared to basal results for kPa and ~4 times greater for the speed of sound (m × s^−1^) at 60% MVC intensity, although significant differences to the basal condition were observed from 10% MVC. Our results are similar to the results of Otsuka, Shan and Kawakami [23], who measured the stiffness of the rectus femoris in dynamic conditions at 60% MVC but without differentiation between muscle regions and obtained a ~4 times increase in the speed of sound (m × s^−1^) at 60% MVC compared to the basal condition. Therefore, our results confirm that shear wave elastography is an applicable technique for evaluating muscle stiffness in dynamic conditions, and following the recommendation of Alfuraih et al. [31], the speed of sound should be the factor of choice for comparison between studies because it is less affected with regard to the under-probe tissue characteristics than kPa values.

The placement of the ROI inside the muscular belly could have a significant influence on the results of the myotendinous stiffness. Otsuka, Shan and Kawakami [23] reported differences between muscle and fascia stiffness evolution during a dynamic evaluation protocol. In addition, the interpretation of muscle stiffness values should not be performed in an isolated manner for muscle groups in which the different muscle bellies may condition the results of the others. Koo, Guo, Cohen and Parker [25] already described an increase in the stiffness of the tibialis anterior due to compression of the anterior crural fascia over the muscle structure, which limits its capacity to increase the thickness during a dorsiflexion ankle movement, which is line with a report by Liu et al. [32], who found an increase in medial gastrocnemius stiffness due to increased tension caused by stretching. In our case, the higher values of muscle stiffness shown by the deep region of the rectus femoris compared to the superficial region may be conditioned by the pressure exerted by the vastus intermedius on the deep aponeurosis of the rectus femoris. In this context, Otsuka, Shan and Kawakami [23], indicated that the muscle contraction increased the stiffness of the fascia lata during the isometric knee extension movement, although this structure was not directly related to the exercise performed. Therefore, future studies should describe the stiffness of analyzed muscles in relation to the structures around them and not in an isolated form.

This work has some limitations. Elastography is a dependent operator technique, so all measurements were made by the same evaluator. In addition, only a specific sample of healthy and young men was included in this study, so it would be necessary to include women in the sample in order to draw conclusions that can be extrapolated to both genders. Finally, the characteristics of the probe used did not allow for recording the stiffness of the patellar tendon when the force exceeded 10% MVC.

## 5. Conclusions

Shear wave elastography application showed a good success percentage for assessing rectus femoris (from relaxed condition to 60% MVC) and patellar tendon stiffness (from relaxed conditions to 10% MVC). At least 20% MVC saturation of the elastogram was described in most of the participants during patellar tendon analysis. The rectus femoris stiffness increased during the incremental isometric contraction test, and inter-regions (superficial vs. deep) differences were found from 30% MVC. This study could be the starting point for future investigations that want to analyze myotendinous stiffness with shear wave elastography in dynamic conditions.

## Figures and Tables

**Table 1:** Mean values of rectus femoris and patellar tendon stiffness (mean ± SD).

Title 1	Young Modulus (kPa)	Speed of Sound (m/s)	Success Percentage (%)
Rectus femoris superficial region			
Basal condition	10.47 ± 2.74	1.91 ± 0.30	100
10% MVC	17.41 ± 8.40	2.50 ± 0.76	100
20% MVC	22.17 ± 10.83	2.78 ± 0.78	100
30% MVC	31.83 ± 17.05	3.27 ± 1.04	100
40% MVC	51.25 ± 28.90	4.11 ± 1.18	95
50% MVC	61.16 ± 37.03	4.59 ± 1.35	95
60% MVC	76.67 ± 36.07	5.09 ± 1.22	85
Rectus femoris deep region			
Basal condition	11.33 ± 4.60	1.93 ± 0.34	100
10% MVC	22.70 ± 18.43	2.65 ± 0.95	100
20% MVC	31.94 ± 26.37	3.11 ± 1.13	100
30% MVC	51.46 ± 38.17 *	3.95 ± 1.42 *	100
40% MVC	75.21 ± 42.27 *	4.83 ± 1.38 *	95
50% MVC	85.34 ± 45.05 *	5.17 ± 1.39	95
60% MVC	109.29 ± 40.04 *	5.84 ± 1.25 *	85
Patellar tendon			
Basal condition	82.37 ± 27.97	5.41 ± 1.56	100
10% MVC	186.24 ± 36.87	6.28 ± 1.83	70
20% MVC	-	-	40
30% MVC	-	-	30
40% MVC	-	-	30
50% MVC	-	-	10
60% MVC	-	-	0

MVC = maximal voluntary contraction; * Difference between superficial and deep muscle regions; *p* < 0.05.

## Data Availability

The data presented in this study are available on request from the corresponding author. The data are not publicly available due to restrictions of the subjects’ agreement.
